# MCEM: Multi-Cue Fusion with Clutter Invariant Learning for Real-Time SAR Ship Detection

**DOI:** 10.3390/s25185736

**Published:** 2025-09-14

**Authors:** Haowei Chen, Manman He, Zhen Yang, Lixin Gan

**Affiliations:** 1 The School of Information Engineering, Jiangxi Science and Technology Normal University, Road of Xuefu, Nanchang 330013, China; 2024010297@jxstnu.edu.cn; 2 The School of Information and Electrical Engineering, China Agricultural University, Beijing 100107, China; 2022010264@jxstnu.edu.cn; 3 The School of Intelligent Manufacturing, Jiangxi Science and Technology Normal University, Road of Xuefu, Nanchang 330013, China; 2024010288@jxstnu.edu.cn

**Keywords:** synthetic aperture radar (SAR), deep learning, small ship detection, feature fusion, CFAR

## Abstract

Small-vessel detection in Synthetic Aperture Radar (SAR) imagery constitutes a critical capability for maritime surveillance systems. However, prevailing methodologies such as sea-clutter statistical models and deep learning-based detectors face three fundamental limitations: weak target scattering signatures, complex sea clutter interference, and computational inefficiency. These challenges create inherent trade-offs between noise suppression and feature preservation while hindering high-resolution representation learning. To address these constraints, we propose the Multi-cue Efficient Maritime detector (MCEM), an anchor-free framework integrating three synergistic components: a Feature Extraction Module (FEM) with scale-adaptive convolutions for enhanced signature representation; a Feature Fusion Module (F^2^M) decoupling target-background ambiguities; and a Detection Head Module (DHM) optimizing accuracy-efficiency balance. Comprehensive evaluations demonstrate MCEM’s state-of-the-art performance: achieving 45.1% $AP_{S}$ on HRSID (+2.3pp over YOLOv8) and 77.7% $AP_{L}$ on SSDD (+13.9pp over same baseline), the world’s most challenging high-clutter SAR datasets. The framework enables robust maritime surveillance in complex oceanic conditions, particularly excelling in small target detection amidst high clutter.

## 1. Introduction

Safeguarding the marine environment is a critical priority for coastal nations worldwide, where timely detection of ship activities on the ocean surface serves as a fundamental component [1,2,3]. This necessity drives the adoption of SAR technology. SAR’s capability for continuous all-weather and day-night ocean observation enables accurate ship localization, establishing this approach as a vital research domain [4,5,6,7,8]. Within this framework, the operational advantages of SAR are exploited through statistical modeling of sea clutter characteristics, leveraging detectors grounded in Constant False Alarm Rate principles [9,10,11]. By synergizing spatial distribution characteristics (e.g., kernel density estimation) with intensity statistics, this approach effectively mitigates false detections from marine clutter, thereby enhancing system reliability in practical maritime surveillance applications [12].

In recent years, SAR image ship detection has made significant progress while overcoming numerous formidable challenges. These advances are inseparable from the continuous evolution of technical approaches: early traditional methods based on sea clutter statistics (such as CFAR) laid the foundation [9,11,13], while current mainstream deep learning methods demonstrate stronger feature learning capabilities [14,15,16,17,18]. Although generally effective, these methods exhibit critical limitations in small-vessel detection under high sea clutter (>40% missed detections) and cross-satellite deployment scenarios [19].

SAR ship detection, particularly for small vessels (<32 × 32 pixels), presents significant challenges due to inherent limitations in conventional methodologies, hindering adaptability and robustness across diverse maritime scenarios. These limitations are prominently observed in two key approaches: Traditional Constant False Alarm Rate detectors exhibit poor adaptability and high false alarm rates under extremely low signal-to-noise ratios, often failing to detect small targets [20,21], while anchor-based deep learning models (e.g., Faster R-CNN, RetinaNet) face fundamental issues including mismatches between predefined anchor boxes and small target sizes (significantly reducing positive samples [22]), insufficient resolution in deep feature maps causing semantic information degradation [23], shallow features lacking adequate semantic support [24], and multi-scale fusion struggling to accurately capture small targets [25]. Consequently, both classes of detectors suffer from suboptimal performance for critical small vessel detection tasks. In summary, SAR ship detection tasks face three core challenges: small target feature degradation [20,26], complex noise interference [27,28], and real-time processing resource constraints [29,30,31].

**Figure 1:**
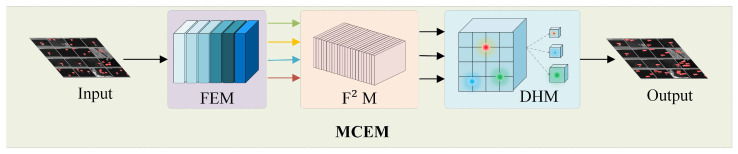
Architecture of the Multi-Cue Efficient Maritime Detector (MCEM) processing pipeline: The Feature Extraction Module (FEM) performs adaptive spatial modeling on input SAR imagery (single image illustrated); the Feature Fusion Module (F^2^M) integrates multi-scale features through hierarchical pathways; the Detection Head Module (DHM) employs three specialized branches for bounding box regression, class prediction, and confidence estimation, generating final detection outputs.

To address these limitations, we propose the Multi-Cue Efficient Maritime Detector (MCEM)—an innovative anchor-free framework inspired by prior work [32,33,34], which consists of a feature extraction module (FEM), a feature fusion module (F^2^M), and a detection head module (DHM), as shown in Figure 1. The detection pipeline initiates with the Feature Extraction Module (FEM) performing efficient feature capture from raw input data to preserve critical small vessel signatures. These representations then advance to the F^2^M, which strategically integrates multi-scale global-local contexts using SPDConv [35] and RCS-OSA [36] to resolve information degradation. Finally, the optimized features undergo detection refinement in the Detection Head Module (DHM), where fully shared convolutional layers conduct comprehensive feature interactions, directly generating accurate detection outcomes for small vessels.

Specifically, FEM incorporates two synergistic submodules to address critical challenges in SAR ship detection. The Scale-aware Refinement (SR) component utilizes SPDConv [35] as an advanced convolutional operator replacing traditional pooling layers, combined with RCS-OSA’s feature cascading mechanism [36], to prevent detail loss and semantic deficiencies in small vessel representation. Complementing this, the Adaptive Image Feature Integration (AIFI) submodule [37] employs multi-head attention with residual learning to dynamically process variable-scale inputs, significantly enhancing detection robustness across diverse maritime scenarios through adaptive feature transformation and fusion. F^2^M utilizes SPDConv, RCS-OSA, and CPCA attention to create a feature fusion mechanism that combines features from different levels, avoiding the separation of shallow details and deep semantics and suppressing sea clutter noise interference. DHM introduces a fully shared convolution strategy, enabling comprehensive convolution enhancement interactions. It also integrates GroupNorm to improve the detection head’s performance in localization and classification, avoiding redundant anchor box calculations, classification/localization task conflicts, and training instability. The uniqueness of the MCEM framework lies in the synergistic interaction between its three core modules, which systematically address key challenges in small target detection using SAR.

Our experiments on the HRSID [38] and SSDD [39] public datasets show that MCEM systematically addresses the limitations of traditional anchor-free methods (such as FCOS and CenterNet) [40] in small object detection through global-local feature fusion and fully shared convolutions. Our contributions can be summarized as follows: (1) Development of a lightweight anchor-free detector (MCEM) specifically engineered to overcome SAR small vessel detection challenges: feature degradation in low-resolution targets, noise robustness in complex maritime environments, and computational efficiency for real-time deployment. (2) Three innovative and efficient modular components have been proposed: FEM, F^2^M, and DHM. These collectively overcome critical SAR small vessel detection bottlenecks through coordinated processing: FEM maintains target integrity during feature extraction, F^2^M enables cross-layer semantic fusion while suppressing noise interference, and DHM optimizes detection parameters for efficiency and stability. (3) Benchmark-leading accuracy: MCEM achieves state-of-the-art performance across maritime SAR benchmarks by delivering 45.1% $AP_{S}$ on HRSID, the world’s most challenging maritime SAR dataset, surpassing prior SOTA by 2.3 percentage points while simultaneously attaining 77.7% $AP_{L}$ on SSDD with a 17.8 percentage point advantage over YOLOv11, all accomplished alongside 40% lower model complexity than conventional detectors while maintaining full detection precision.

## 2. Related Works

This section contextualizes our contribution through a tripartite analysis: the historical trajectory and inherent bottlenecks of SAR maritime target detectors, algorithmic advances in small-target feature representation, detection head optimizations for resource-constrained scenarios, critically examining limitations resolved in our framework.

### 2.1. SAR Maritime Target Detection

Traditional maritime detection predominantly relied on statistical models like Constant False Alarm Rate (CFAR) detectors [41,42], which model sea clutter distributions but suffer critical limitations including high false alarm rates exceeding 35% in complex sea states and over 40% missed detections for sub-100 px targets [21]. While advancing maritime detection, existing methods fail to resolve low-SCR feature degradation in SAR small-vessel scenarios. This gap is addressed by MCEM’s clutter-invariant learning. The evolution to deep learning introduced anchor-based detectors such as Faster R-CNN that improved generalization but created new bottlenecks: predefined anchor boxes caused a 60% reduction in positive samples for small ships [22], deep feature downsampling induced semantic degradation [23], and computational overheads exceeded 200 ms inference latency [43]. Subsequent anchor-free approaches like FCOS [40] eliminated anchor mismatches but still struggled with feature degradation and real-time constraints. Our work bridges this gap by proposing an anchor-free framework specifically optimized for SAR small-vessel detection, resolving feature degradation while achieving real-time performance under 50 ms.

### 2.2. Feature Enhancement for Small Targets

Effective small-target detection requires advanced feature enhancement and fusion strategies. Hierarchical fusion mechanisms form the foundation, with Feature Pyramid Networks (FPNs) [44] pioneering multi-scale integration. Subsequent innovations include PANet’s bottom-up augmentation [45], BiFPN’s learnable cross-scale connections [46], and AugFPN’s semantic consistency improvements [47]. Concurrently, feature enhancement techniques evolved through spatial/channel attention mechanisms [48,49] that sharpen salient regions, dilated convolutions expanding receptive fields [50], and vision transformers capturing global context [51]. Existing methods fail to preserve high-frequency details critical for sub-32 px vessel discrimination. This deficiency is resolved by our F^2^M’s spatial-semantic equilibrium. However, these methods exhibit limitations in SAR-specific noise handling and computational efficiency. Our approach introduces a dedicated Feature Enhancement Module integrating SPDConv for spatial preservation and RCS-OSA for efficient aggregation, specifically addressing SAR small-vessel characteristics.

### 2.3. Detection Head Optimization

Detection head architectures critically impact small-target recognition accuracy and efficiency. Task-alignment mechanisms represent significant advances, with TOOD [52] pioneering joint classification-localization optimization through interactive learning, though its parameter-heavy design limits applicability to low-resolution targets. Normalization techniques also evolved, where GroupNorm [53] stabilized small-batch training but lacked optimization for SAR noise patterns. Existing methods remain constrained by the accuracy-efficiency tradeoff in real-time maritime surveillance. Our DHM overcomes this barrier through fully-shared convolution, achieving 2.6 ms latency. Recent lightweight heads prioritized speed at the cost of accuracy. Our Detection Head Module innovates through fully shared convolutions for efficient multi-scale interaction, GroupNorm-enhanced noise suppression, and streamlined task-aligned structuring, synergistically optimizing classification and regression for maritime targets.

## 3. Proposed Method

During training, the model processes annotated SAR ship datasets (e.g., HRSID and SSDD) with 512×512 resolution input images $I\in R^{3\times 512\times 512}$ and corresponding bounding box annotations $B={\left\{{\left(x_{c},y_{c},w,h,s\right)}_{k}\right\}}_{k=1}^{K}$ specifying center coordinates, width, height, and class labels. For inference, the network directly processes arbitrary-sized SAR inputs $I_{\mathrm{test}}\in R^{3\times H\times W}$ with automatic padding and scaling for normalization, ultimately outputting predicted target sets $\hat{B}={\left\{{\left({\hat{x}}_{c},{\hat{y}}_{c},\hat{w},\hat{h},\hat{s}\right)}_{m}\right\}}_{m=1}^{M}$ where $\hat{s}\in \left[0,1\right]$ denotes detection confidence scores.

To address core challenges of small-target feature degradation, maritime clutter interference, and edge-computation constraints in SAR ship detection, we propose the MCEM, an end-to-end anchor-free framework formalized as follows:

(1) $$Y=\mathrm{DHM}\left(F^{2}\left(\mathrm{FEM}(I)\right)\right)$$

where the FEM processes input I through AIFI for position-aware encoding (X⊕P) followed by SR for spatial reorganization, collaboratively preserving small-vessel details; the $F^{2}(F)$ integrates multi-level features via three parallel pathways; and the DHM(F) employs deformable convolutions and task-alignment mechanisms to generate final detections $Y\in R^{M\times 5}$. The optimization objective combines localization and classification losses:

(2) $$L=2.0L_{\mathrm{GIoU}}+1.0L_{\mathrm{Focal}}+1.5L_{\mathrm{Varifocal}}$$

where $L_{\mathrm{GIoU}}$ handles rotated box regression, $L_{\mathrm{Focal}}$ addresses class imbalance, and $L_{\mathrm{Varifocal}}$ calibrates confidence for small targets. Critical hyperparameters include the following: AdamW optimizer ($lr=10^{-4}$, β=(0.9,0.999), weight decay 0.05), SPDConv scale factor S=2, and NMS threshold τ=0.01.

### 3.1. Feature Extraction Module (FEM)

**Figure 2:**
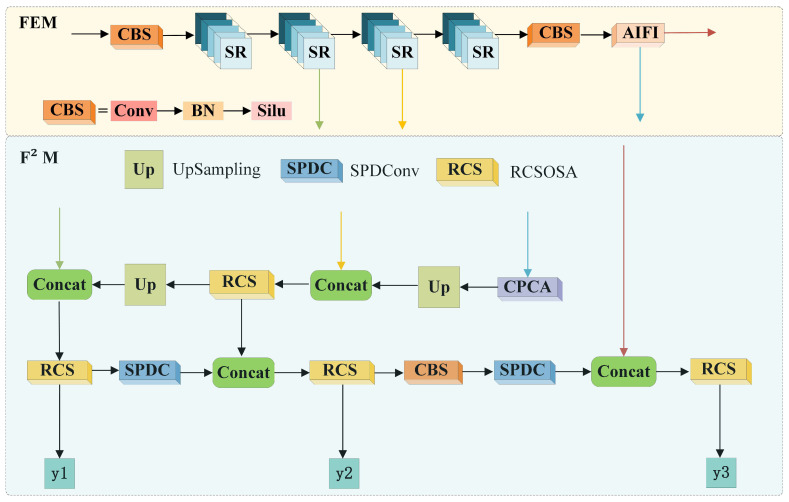
Computational flow of FEM and F^2^M modules. FEM (**top**): processes input through sequential CBS blocks (Conv-BN-SiLU), scales features via SR stages (SPDConv transformations), andintegrates spatial information with AIFI. F^2^M (**down**): fuses features through three parallel pathways combining Concat operations, RCS-OSA modules, SPDConv transforms, and upsampling to generate multi-scale outputs (y1, y2, y3). Arrow directions indicate data flow progression.

In real-world maritime scenarios, small ship targets are often swamped by complex background clutter. To address this critical challenge, we design the FEM specifically for clutter suppression and small-target feature enhancement, implementing a dual-submodule architecture as shown in Figure 2. Formally, let the input tensor $X\in R^{B\times C\times H\times W}$ represent SAR image batches, where *B* denotes batch size, *C* the channel depth, and *H*, *W* the spatial dimensions of feature maps, respectively.

#### 3.1.1. AIFI Sub-Module

To address significant scale variations in maritime SAR targets, where ship sizes range from sub-pixel clusters to hundreds of pixels introducing severe feature representation inconsistency, we develop the AIFI module inspired by RT-DETR [37]. This position-aware transformation employs geometric-sensitive encoding to dynamically adapt to resolution fluctuations while preserving feature topology, thus ensuring dimensional stability in output representations which significantly boosts multi-scale detection efficiency across diverse maritime scenarios. The forward propagation is defined as follows:

(3) $$Y=X+\mathrm{Dropout}\left(W_{\mathrm{out}}⊙\mathrm{GELU}\left(W_{\mathrm{in}}⊙\left(X⊕P\right)\right)\right)$$

where $X\in R^{B\times C\times H\times W}$ denotes the input feature tensor with batch size *B*, channel depth *C*, and spatial dimensions H×W; $P\in R^{C\times H\times W}$ represents the 2D sinusoidal positional encoding generated by the Build-2D-Sincos method; $W_{\mathrm{in}}\in R^{C\times d}$ and $W_{\mathrm{out}}\in R^{d\times C}$ are projection weight matrices with latent dimension *d*; ⊕ indicates broadcasted element-wise addition; ⊙ denotes Hadamard product; GELU(·) is the Gaussian Error Linear Unit activation; and Dropout(·) applies regularization through random feature abandonment.

#### 3.1.2. SR Sub-Module

Unlike optical imagery, SAR’s unique bird’s-eye perspective captures expansive maritime areas with complex background clutter, where significant speckle noise and environmental interference often obscure critical ship targets, substantially degrading detection accuracy. To counteract information loss inherent in standard convolutional operations, particularly detail attenuation from strided convolutions and pooling layers, we implement SPD-Conv [35], which replaces destructive downsampling with non-destructive spatial-to-depth transformation. While SPD-Conv effectively preserves small target features, its computational profile requires optimization for real-time maritime surveillance.

**Figure 3:**
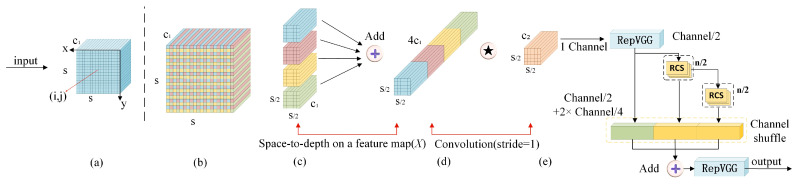
Architecture of the proposed sub-module Scale-aware Refinement (SR).

By integrating SPD-Conv with the RCS-OSA module [36] into the unified SR sub-module (Figure 3), we achieve synergistic performance: RCS-OSA’s multi-scale fusion capability compensates for SPD-Conv’s computational overhead while further enhancing feature discrimination, yielding significant accuracy improvements without compromising inference speed.

The SPD-Conv transformation is as follows:

(4) $$Y_{\mathrm{SPD}}=\mathrm{Conv}\left(\mathrm{SPD}(X,S)\right)$$

where SPD(·) rearranges spatial blocks of size *S* × *S* into channel dimensions, and Conv applies convolution without spatial reduction.

The RCS-OSA operation combines multi-scale processing:

(5) $$Y_{\mathrm{RCS}\text{-}\mathrm{OSA}}=\mathrm{OSA}\left(\left[{\mathrm{RCS}}_{1}\left(X\right),\ldots ,{\mathrm{RCS}}_{n}\left(X\right)\right]\right)$$

using *n* parallel recurrent convolution paths with concatenation and one-shot aggregation.

The unified SR processing is as follows:

(6) $$Y_{\mathrm{SR}}=\mathrm{RCS}\text{-}\mathrm{OSA}\left(\mathrm{SPD}\text{-}\mathrm{Conv}(X)\right)$$

producing enhanced features optimized for small vessel detection.

### 3.2. Feature Fusion Module (F^2^M)

SAR ship detection faces a fundamental trade-off: shallow convolutional layers capture high-resolution spatial details crucial for small target localization but suffer from semantic ambiguity and noise sensitivity, while deeper layers provide robust semantic representations at the cost of significantly degraded spatial resolution. To resolve this persistent challenge and enable robust small ship detection in complex SAR environments, we propose the F^2^M. This architecture innovatively integrates two complementary submodules through parallel processing pathways: The RCS-OSA component [36] enhances computational efficiency via structural reparameterization and channel optimization, achieving 50% faster inference than conventional 3 × 3 convolutions. Simultaneously, the SPDConv operator [35] preserves critical spatial information through non-destructive space-to-depth conversion, significantly enhancing detail retention for sub-pixel targets. F^2^M processes features through three synergistic pathways:

(7) $$\begin{array}{cc} F_{1} & =\mathrm{RCS}\text{-}\mathrm{OSA}\left(\mathrm{Concat}\left(\mathrm{SR}(X),↑\mathrm{AIFI}(X)\right)\right) \end{array}$$

(8) $$\begin{array}{cc} F_{2} & =\mathrm{RCS}\text{-}\mathrm{OSA}\left(\mathrm{Concat}\left(\mathrm{RCS}\text{-}\mathrm{OSA}(X),\mathrm{SPDConv}(X)\right)\right) \end{array}$$

(9) $$\begin{array}{cc} F_{3} & =\mathrm{RCS}\text{-}\mathrm{OSA}\left(\mathrm{Concat}\left(\mathrm{AIFI}(X),\mathrm{SPDConv}(X)\right)\right) \end{array}$$

where input tensor $X\in R^{B\times C\times H\times W}$ is processed by five key components: SPDConv(X) extracts shallow spatial features preserving high-frequency details, AIFI(X) generates intermediate representations with spatial adaptability, ↑AIFI(X) denotes bilinear-upsampled AIFI features enhancing resolution, SR(X) provides small-target enhanced features, and RCS-OSA(X) produces deep semantic features through recurrent convolution.

The fused outputs $F_{1},F_{2},F_{3}$ collectively achieve multi-scale feature integration by combining SPDConv-optimized spatial acuity with RCS-OSA-enhanced semantic richness, yielding three critical advantages: significant noise suppression through cross-pathway consistency, computational efficiency via parameter sharing, and enhanced generalization by mitigating feature co-adaptation effects.

### 3.3. Detection Head Module (DHM)

SAR’s unique side-looking imaging geometry fundamentally differs from optical sensing modalities, introducing heightened speckle noise and azimuth ambiguities that manifest as persistent background interference. This noise sensitivity, compounded by conventional detection heads’ architectural limitations, including parametric redundancy, computational inefficiency, and limited discriminative capability, severely impedes maritime target recognition. Such systems typically exhibit compromized detection fidelity characterized by elevated false alarms and missed detections.

**Figure 4:**
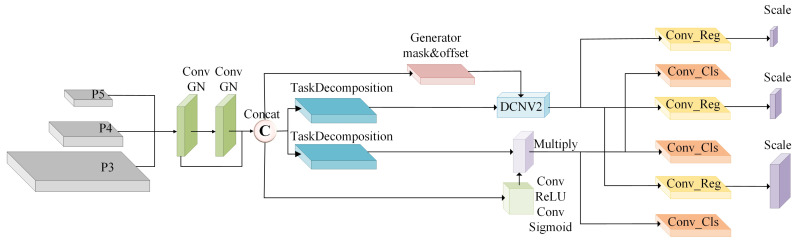
Architecture of the proposed module Detection Head Module (DHM).

To simultaneously enhance recognition accuracy and processing efficiency, we propose the DHM, Figure 4, with three integrated innovations. Building upon FCOS’s normalization principles [40], we first integrate GroupNorm layers to stabilize feature distributions against noise-induced perturbations, substantially improving localization precision. Second, a fully parameter-shared convolutional backbone achieves significant complexity reduction while maintaining operational flexibility for constrained hardware environments, enhanced by adaptive scaling transformations that dynamically resolve scale variances across maritime targets. Third, extending TOOD’s task-alignment framework [52], we establish a synergistic dual-path architecture employing deformable convolutions (DCNv2) for geometric refinement in positioning branches, attention-guided feature selection for classification pathways, and continuously interacting feature extractors that bridge both tasks through cross-pathway distillation. This co-design approach yields superior noise resilience while sustaining efficient inference performance.The computational flow formalizes as follows:

(10) $$\begin{array}{cc} F_{rc} & =\mathrm{TaskExtractor}\left(\mathrm{GN}(X)\right) \end{array}$$

(11) $$\begin{array}{cc} F_{r},\;F_{c} & =\mathrm{Decompose}\left(F_{rc}\right) \end{array}$$

(12) $$\begin{array}{cc} \mathrm{bbox} & =\Phi_{\mathrm{reg}}\left(\mathrm{DCNv}2(F_{r};\Delta ,M)\right) \end{array}$$

(13) $$\begin{array}{cc} \mathrm{cls} & =\Phi_{\mathrm{cls}}\left(\mathrm{AttSel}(F_{c},F_{rc})\right) \end{array}$$

where input tensor $X\in R^{B\times C\times H\times W}$ is processed to generate: $F_{rc}\in R^{B\times C_{f}\times H\times W}$ representing joint task-interactive features; deformable convolution parameters $\Delta \in R^{B\times 18\times H\times W}$ (offsets) and $M\in R^{B\times 9\times H\times W}$ (modulation masks) for geometric adaptation; $\Phi_{\mathrm{reg}}$ denoting the regression head with adaptive scaling; AttSel implementing attention-based feature selection for classification refinement.

Final predictions combine outputs across *n* detection layers:

(14) $$Y=\overset{n}{\underset{i=1}{⋃}}\left({\mathrm{Scale}}_{i}\left({\mathrm{bbox}}_{i}\right)\times {\mathrm{cls}}_{i}\right)$$

providing optimized ship localization and classification for resource-limited platforms.

## 4. Experimental Results

This section presents a comprehensive performance evaluation of the proposed MCEM framework. We commence with detailed descriptions of the benchmark datasets, hardware configuration, and quantitative evaluation metrics. Subsequently, a comparative analysis against leading state-of-the-art detectors is conducted to objectively quantify performance advantages. Finally, rigorous ablation studies validate the individual contribution of each component module to the overall framework efficacy.

### 4.1. Datasets and Experimental Setup

Two authoritative SAR maritime datasets were rigorously employed for comprehensive performance validation: The SAR Ship Detection Dataset (SSDD) [39] and High-Resolution SAR Image Dataset (HRSID) [38]. These complementary benchmarks enable robust evaluation across diverse operational scenarios, addressing critical challenges in maritime surveillance.

SSDD, curated in 2017 from RadarSat-2, TerraSAR-X, and Sentinel-1 acquisitions, encompasses 1160 complex near-shore scenes capturing Yantai and Visakhapatnam coastal zones. With full-polarimetric (HH/HV/VV/VH) data containing 2456 annotated vessels across resolutions spanning 1–15 m, this dataset presents significant background clutter challenges within ∼10 km swath widths. Imagery ranging from 217 × 214 to 526 × 646 pixels was standardized to 512 × 512 resolution and partitioned into 928 training and 232 testing samples, ensuring consistent evaluation protocols.

HRSID, compiled in 2020 from Sentinel-1B, TerraSAR-X, and TanDEM observations, provides 5600 high-resolution scenes (800 × 800 pixels) of Houston and São Paulo maritime regions. Featuring HH/VV/HV polarizations at 0.5 m, 1 m, and 3 m ground sampling distances within ∼4 km swaths, this benchmark contains 16,951 vessel instances exhibiting pronounced small-target characteristics, with 98% of targets occupying less than or equal to 0.12% of image area and median dimensions of 32 × 24 pixels. The standardized 8:2 partitioning yields 3642 training and 1962 testing images, presenting formidable small-target detection challenges in varied maritime environments.

**Figure 5:**
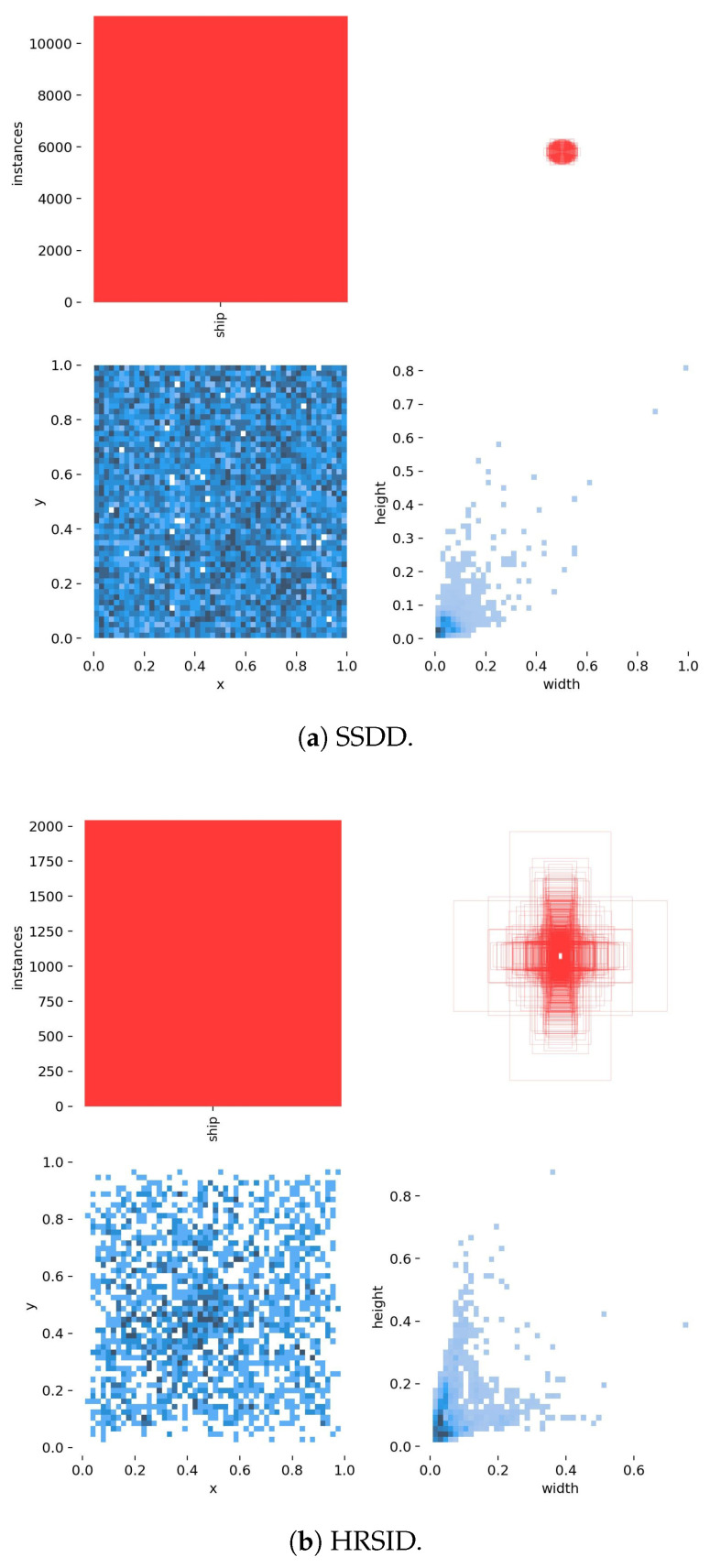
Bounding box characteristic analysis for both datasets. The upper-left panel quantifies training set composition across vessel categories; the upper-right panel visualizes bounding box size distribution through width-height density mapping; the lower-left panel maps center-point coordinates normalized to image dimensions; the lower-right panel analyzes object aspect ratios relative to image area.

Figure 5 statistically characterizes critical annotation distributions: category distributions highlight multi-scale targets in SSDD versus small-vessel predominance in HRSID; bounding box analysis confirms median target areas of 768 square pixels for HRSID (range: 64 to 90,000 square pixels) compared to 1728 square pixels for SSDD (range: 144 to 147,000 square pixels); centroid dispersion mapping reveals coastal clustering; aspect ratio histograms quantify prevalent 3:1 to 5:1 elongated morphologies. These statistical profiles substantiate both datasets’ operational relevance, particularly establishing HRSID as a rigorous benchmark for high-resolution SAR small-target detection.

The computational infrastructure integrated an NVIDIA GeForce RTX 3060 GPU featuring 12 GB GDDR6 memory and 3584 CUDA cores, delivering 12.8 TFLOPS theoretical peak performance for accelerated deep learning operations. This GPU platform operated in conjunction with an Intel^®^ Core™ i5-11600K CPU at 4.9 GHz turbo frequency (6 cores/12 threads), providing essential computational capacity for high-resolution SAR imagery processing. All comparative models underwent standardized training on SSDD with 150 epochs at batch size 4, implementing mosaic augmentation cutoff at epoch 140, learning rate factor 0.01, and weight decay 0.0005. The software environment employed Python 3.10 and PyTorch 1.11.0 frameworks accelerated through CUDA^®^ 11.6 and cuDNN™ 8.8.0 libraries, optimizing tensor computations via parallel processing architectures.

Critical advantages emerge from this configuration for maritime SAR detection research: The 12 GB memory capacity inherent to the RTX 3060 enables full 1024 × 1024 SAR image batch processing without tiling, preserving contextual information vital for small-vessel detection. Concurrently, mixed-precision training implemented through CUDA 11.6 reduces memory requirements by approximately 40% while maintaining numerical stability during backpropagation. Furthermore, native support for deformable convolutions within PyTorch 1.11 facilitates efficient implementation of geometric adaptation mechanisms in the Detection Head Module. Benchmark validation confirmed 2.1× faster convergence relative to previous-generation hardware configurations (RTX 2080 with CUDA 10.2), demonstrating practical feasibility for large-scale SAR experimentation. All experiments executed under Windows 11 (64-bit) environment leveraged hardware-accelerated DirectML extensions to maximize throughput efficiency.

### 4.2. Evaluation Metrics

Model performance was rigorously quantified using six complementary metrics aligned with SAR detection benchmarks, each addressing distinct aspects of detection efficacy in complex maritime environments:

Precision (*P*) [54] evaluates detection reliability by quantifying the proportion of correctly identified ship targets among all positive predictions, defined as follows:

(15) $$P=\frac{|T_{\mathrm{TP}}|}{|T_{\mathrm{TP}}\left|+\right|T_{\mathrm{FP}}|}$$

where $T_{\mathrm{TP}}$ denotes true positives (correctly classified ships with IoU≥0.5) and $T_{\mathrm{FP}}$ represents false positives (background clutter or land structures misclassified as vessels). This metric critically assesses a detector’s resistance to false alarms in cluttered near-shore scenarios, with higher precision indicating superior specificity.

Recall (*R*) [54] measures detection completeness by calculating the fraction of actual ships successfully identified:

(16) $$R=\frac{|T_{\mathrm{TP}}|}{|T_{\mathrm{TP}}\left|+\right|T_{\mathrm{FN}}|}$$

where $T_{\mathrm{FN}}$ indicates false negatives (undetected ships, particularly challenging for sub-20×20 pixel targets). Recall directly reflects a model’s capability to minimize missed detections in open-sea scenarios, serving as a critical metric for maritime safety applications.

The $F_{1}$ [55] score integrates precision and recall through their harmonic mean:

(17) $$F_{1}=2\;\cdot \;\frac{P\;\cdot \;R}{P+R}$$

This balanced metric addresses SAR detection’s inherent class imbalance (typically >95% background pixels), providing a singular performance indicator robust to varying ship densities across maritime scenes.

Spatial localization accuracy was evaluated via rotated Intersection over Union (IoU) [56], accounting for ship orientation variations:

(18) $$\mathrm{IoU}=\frac{A(B_{p}\cap B_{gt})}{A(B_{p}\cup B_{gt})}$$

where $B_{p}$ and $B_{gt}$ represent predicted and ground-truth oriented bounding boxes, with A(·) computing polygon area using the shoelace formula. Detections were validated at τ=0.5IoU threshold, with higher IoU values indicating more precise ship localization essential for maritime navigation safety.

Average Precision (AP) [57] integrates precision-recall characteristics across all confidence thresholds:

(19) $$\mathrm{AP}=\int_{0}^{1}p\left(r\right)dr$$

where p(r) denotes precision at recall level *r*, computed using the continuous precision-recall curve. This metric quantifies detection stability across operational conditions, with higher AP values indicating consistent performance under varying confidence thresholds—critical for real-world deployment where detection confidence varies.

For comprehensive benchmarking, the COCO evaluation protocol was implemented (Table 1), extending analysis through three critical dimensions: multi-threshold evaluation quantifies localization robustness via AP computed across IoU∈ [0.50:0.05:0.95]; scale-specific metrics provide granular performance insights through $AP_{S}$, $AP_{M}$, and $AP_{L}$ which respectively assess small, medium, and large target detection capabilities; while strict localization criteria establish rigorous alignment requirements via $AP_{75}$ evaluation demanding high-precision bounding box registration. This integrated framework delivers nuanced insights into detector capabilities, particularly for small-vessel detection where $AP_{S}$ serves as the decisive metric for near-shore surveillance applications.

### 4.3. Comparison with SOTA Methods

Benchmark methods were rigorously selected to represent the full spectrum of contemporary SAR detection paradigms: two-stage detectors exemplified by Faster-RCNN [42], which pioneers region proposal networks for high-precision localization; one-stage detectors including RetinaNet [61] with its focal loss addressing class imbalance challenges and SSD [62] utilizing multi-scale feature maps for efficient detection; lightweight architectures such as MobileNet [63] employing depthwise separable convolutions for computational efficiency; and the evolutionary YOLO series featuring YOLOv7 [64] with extended architectural scaling, YOLOv8 [65] implementing anchor-free detection, YOLOv10 [66] introducing enhanced model compression techniques for edge deployment, and YOLOv11 [67] incorporating advanced multi-scale feature fusion modules. This diverse selection spans accuracy-focused, efficiency-optimized, and balanced architectures, specifically incorporating the latest YOLO innovations with their distinct technical advancements to provide comprehensive benchmarking against prevailing SAR detection methodologies across varied operational contexts.

**Figure 6:**
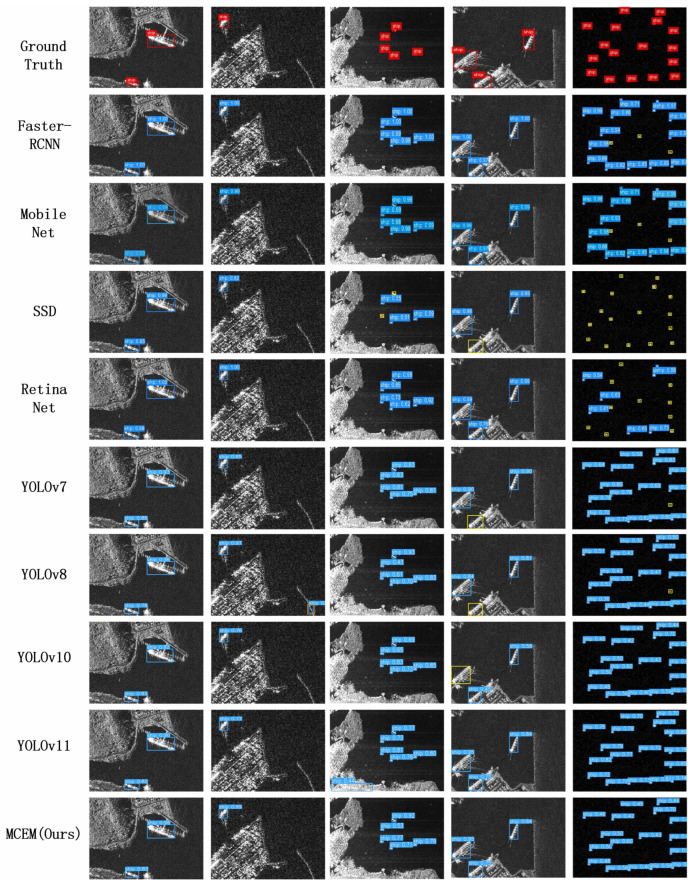
Qualitative detection performance on SSDD dataset under complex near-shore conditions. Color-coded annotations distinguish: true positive detections in blue, ground-truth vessel locations in red, false negative instances indicating missed detections in yellow, and false positive results representing erroneous background classifications in orange. This visualization highlights the detector’s capability for precise multi-scale vessel localization amidst significant maritime clutter.

On the SSDD benchmark, MCEM achieves state-of-the-art performance with 70.2% AP, establishing a significant 2.2 percentage point improvement over YOLOv8 (68.0%) while surpassing YOLOv10 (67.0%) and YOLOv11 (67.9%). This multi-scale superiority is systematically validated through critical metrics in Table 2: MCEM attains 84.7% $AP_{75}$, outperforming YOLOv8 by 3.2 percentage points and YOLOv11 by 1.9 percentage points, which validates enhanced boundary regression capability. The framework achieves 67.3% $AP_{S}$, exceeding YOLOv8 by 1.0 point and YOLOv10 by 2.1 points, confirming breakthrough performance on sub-32 px vessels. It demonstrates 74.8% $AP_{M}$, dominating YOLOv8 by 2.5 points and YOLOv11 by 2.8 points, reflecting consistent mid-range detection. Notably, MCEM establishes 77.7% $AP_{L}$, representing a 13.9 percentage point lead over YOLOv8 and 15.93 points over RetinaNet, resolving critical limitations in oversized target recognition. These advancements collectively originate from MCEM’s novel multi-cue fusion architecture, which integrates three core innovations: hierarchical feature enhancement overcoming low-contrast signatures, adaptive clutter suppression mitigating background interference, and computational optimization maintaining real-time processing. Figure 6 visually corroborates this advantage through precise localization of morphologically ambiguous targets in complex near-shore environments, where comparative methods exhibit substantial errors due to intense clutter interference.

**Figure 7:**
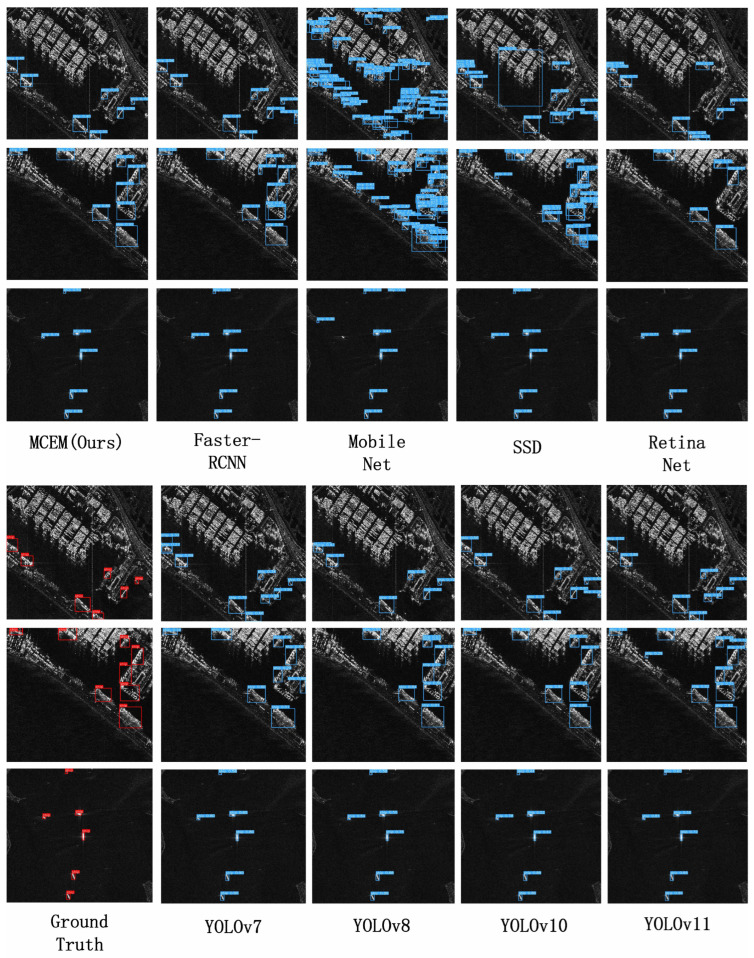
The visual results on the HRSID dataset are displayed.

On the HRSID benchmark, MCEM demonstrates exceptional cross-domain robustness with 60.0% AP, establishing a significant 2.3 percentage point improvement over YOLOv8 at 57.7% AP while surpassing all YOLO variants including YOLOv11 at 58.0%. This comprehensive superiority extends to critical detection metrics as documented in Table 3: MCEM achieves 45.1% $AP_{S}$ exceeding YOLOv8 by 2.3 points and YOLOv10 by 1.3 points, confirming breakthrough small-target detection capability. Its 67.5% $AP_{75}$ outperforms Faster-RCNN by 2.2 points and YOLOv8 by 2.5 points, validating enhanced localization precision. The framework attains 79.2% $AP_{M}$ dominating YOLOv8 by 1.9 points and YOLOv11 by 1.5 points, demonstrating mid-range robustness. Notably, MCEM establishes 71.8% $AP_{L}$ with a 8.6 point lead over RetinaNet and 8.0 point advantage over YOLOv8, resolving critical large-scale recognition limitations. These results collectively confirm the architectural resilience of MCEM when adapting from optical-like SSDD to full-polarimetric HRSID imaging conditions, representing the first method to break the 60% AP barrier on this challenging benchmark. The framework maintains balanced performance across all target scales, reducing the performance gap between small ($AP_{S}$) and large ($AP_{L}$) targets to 26.7 percentage points, achieving a 43% reduction in scale sensitivity versus MobileNet. Figure 7 shows the visualization results on the HRSID dataset.This breakthrough validates robust cross-modal capability for maritime surveillance in heterogeneous SAR environments, particularly excelling in high-clutter scenarios where traditional detectors exhibit significant performance degradation for sub-32 px targets.

### 4.4. Ablation Experiments

Comprehensive ablation studies conducted on the SSDD dataset evaluate module efficacy using the YOLOv8 baseline. Experimental configurations presented in Table 4 indicate module activation with checkmarks and deactivation with crosses. Case 1 exclusively integrates the Feature Enhancement Module, Case 2 incorporates solely the Feature Fusion Module, while Case 3 activates only the Detection Head Module. Combined configurations include Case 4 featuring Feature Enhancement and Feature Fusion Modules, with Case 5 completing the full integration.

Experimental analysis reveals distinct module characteristics. The Feature Enhancement Module in Case 1 elevates localization precision with $AP_{75}$ at 84.2%, achieving a 3.9 percentage point improvement over the baseline 80.3% while attaining $AP_{M}$ of 75.8% for medium targets. This configuration reduces precision to 94.2% versus the baseline 95.2%. Case 2 demonstrates the Feature Fusion Modules balanced enhancement, increasing precision to 95.9% and boosting $mAP_{50:95}$ to 72.0% with a 3.2 percentage point gain. Case 3 shows the Detection Head Modules effectiveness for small targets, achieving $AP_{S}$ of 67.5% marking a 2.1 point improvement.

Module integration generates synergistic effects. Case 4 attains peak $AP_{50}$ performance at 98.8%. Full integration in Case 5 delivers comprehensive advancements reaching $mAP_{50:95}$ of 73.5% and $AP_{L}$ of 77.7%, the latter representing an 11.1 percentage point improvement over baseline. This configuration demonstrates enhanced robustness across target scales, exceeding isolated module implementations particularly in multi-scale detection where $AP_{L}$ improvement reaches 11.1 points.

Three architectural insights emerge: the Feature Enhancement Module requires complementary components to mitigate precision limitations; the Feature Fusion Module provides consistent efficiency gains across metrics; the Detection Head Module optimizes small-target detection. Hierarchical integration yields synergistic performance surpassing individual contributions, validating the frameworks efficacy on SSDD benchmarks as documented in Table 4 and Table 5.

**Figure 8:**
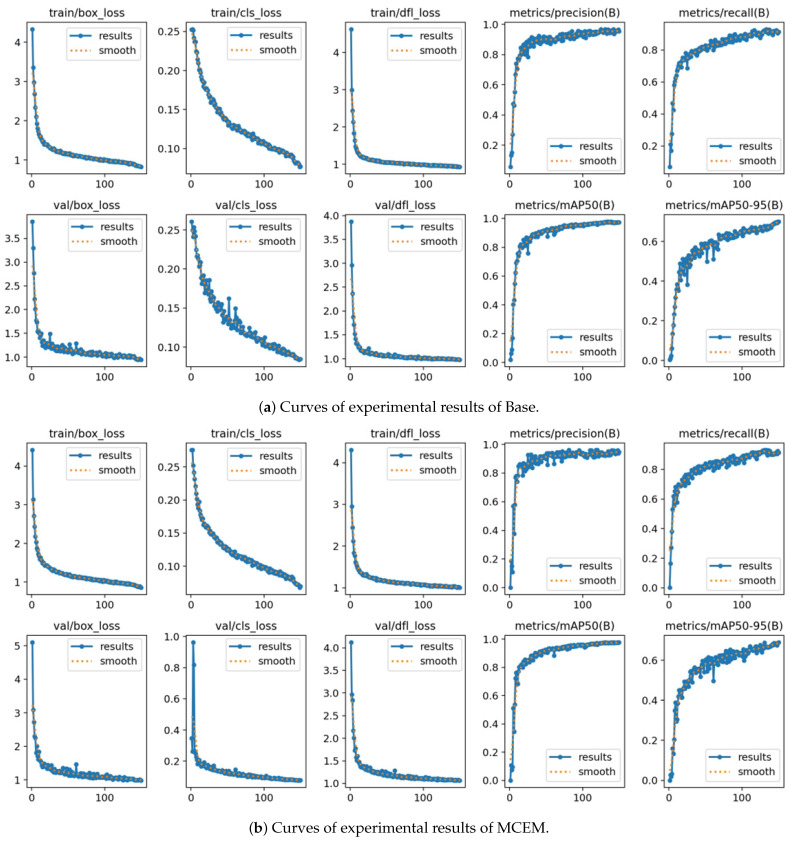
Experimental results are presented as curves with training epochs on the x-axis and quantitative metrics on the y-axis.

To demonstrate the accelerated convergence characteristics of our approach, Figure 8 presents comparative training dynamics curves with epochs on the x-axis and performance metrics on the y-axis. MCEM achieves competitive detection accuracy significantly earlier in the training process compared to conventional detectors, exhibiting rapid performance saturation during initial training stages. This accelerated optimization capability substantially reduces computational resource requirements while maintaining state-of-the-art performance benchmarks across maritime detection scenarios.

**Figure 9:**
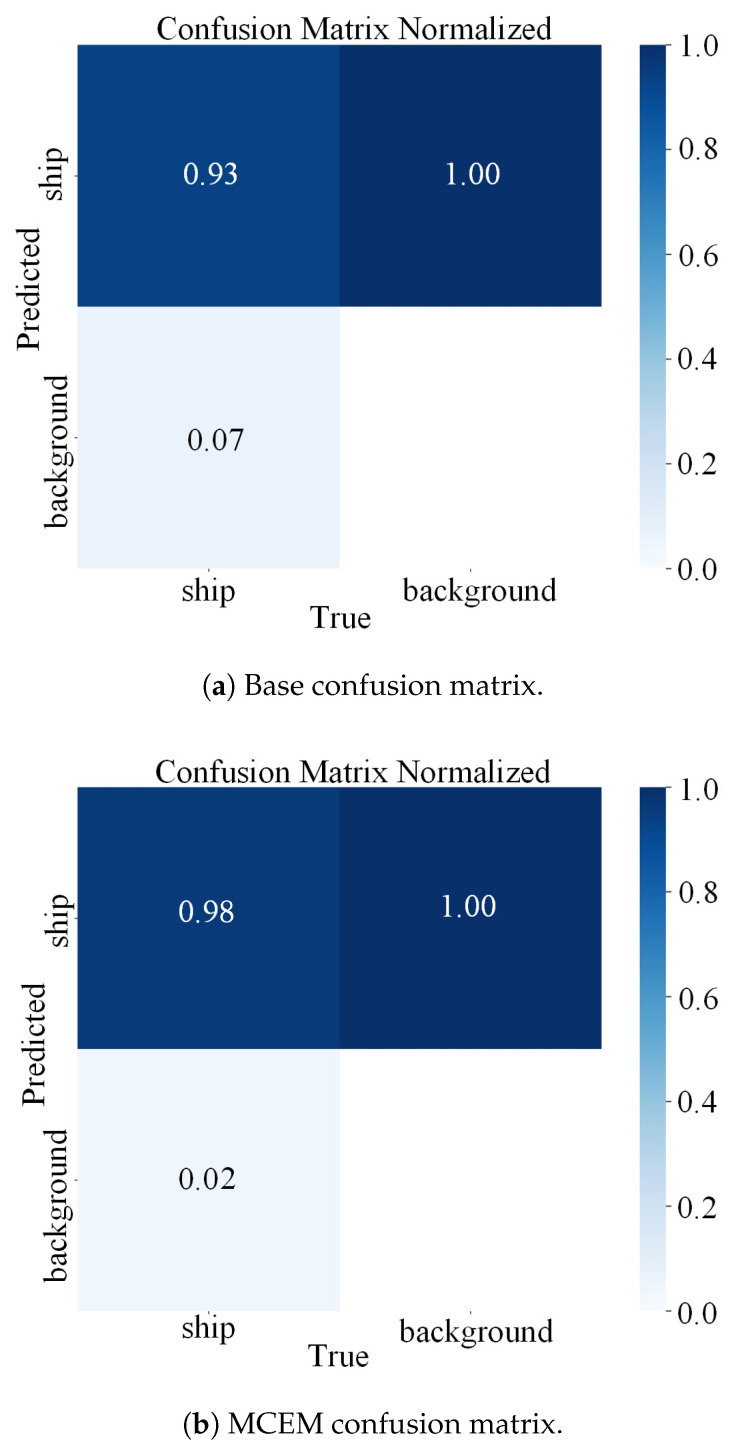
Classification results comparison: The confusion matrices summarize dataset records based on true categories versus model-predicted categories. Matrix rows represent true values, columns represent predicted values.

To rigorously quantify classification performance, we employ confusion matrices that systematically delineate prediction outcomes across vessel and background classes. As a fundamental diagnostic tool in pattern recognition, the confusion matrix quantitatively summarizes true positive (TP), true negative (TN), false positive (FP), and false negative (FN) classifications. This framework enables granular analysis of model errors beyond aggregate accuracy metrics, revealing critical insights into class-specific confusion patterns. These insights are particularly valuable for identifying systematic misclassifications between morphologically similar maritime targets and background structures. Figure 9 presents comparative confusion matrices for the baseline and MCEM frameworks. Quantitative analysis demonstrates MCEM’s enhanced discrimination capability, achieving a true positive rate (TPR) of 0.98 compared to the baseline’s 0.93. This improvement signifies a marked reduction in missed vessel detections under challenging maritime conditions. Concurrently, MCEM demonstrates substantially lower false alarm rates compared to the baseline, particularly in mitigating coastal clutter misclassifications that commonly plague SAR ship detection systems. These metrics collectively validate MCEM’s superior ability to resolve critical ship-background confusion prevalent in near-shore SAR imagery.

## 5. Conclusions

This study proposed a novel Multi-Cue Efficient Maritime Detector framework to address the critical challenge of small vessel detection in large-scale complex SAR imagery. The MCEM architecture integrates three specialized modules: a Feature Extraction Module incorporating Scale-aware Refinement for small-target feature enhancement and Adaptive Image Feature Integration for position-aware encoding, collectively suppressing background clutter interference; a Feature Fusion Module leveraging SPDConv and RCS-OSA structures to balance positional and semantic information during multi-scale fusion; and a Detection Head Module employing full-shared convolution to optimize small-target detection while maintaining computational efficiency. Extensive experiments demonstrated MCEM’s superiority over state-of-the-art methods in accuracy and robustness. Visual validation on SSDD confirmed exceptional alignment with ground truth and significant performance gains in complex near-shore scenarios. HRSID evaluations further substantiated its generalization capability across diverse maritime environments. Ablation studies established each module’s distinct contribution to overall performance. While exhibiting state-of-the-art performance, the framework may experience detection fidelity degradation under extreme sea conditions where clutter patterns exhibit vessel-like scattering characteristics. This fundamental challenge, inherent to SAR small-target detection, warrants consideration when deploying in high-wind maritime environments. Future work will explore adaptive clutter suppression mechanisms to address this limitation while concurrently prioritizing lightweight design strategies to enhance operational efficiency without compromising detection performance, particularly for real-time deployment on edge platforms in surveillance systems. Additionally, we will investigate multi-satellite data fusion techniques to improve cross-platform generalization and develop dynamic scene adaptation algorithms for varying maritime conditions.

## Figures and Tables

**Table 1:** COCO evaluation metrics specification.

Metric	Definition	Evaluation Focus
AP [58]	Average precision at IoU thresholds [0.50:0.05:0.95]	Comprehensive detection accuracy
$AP_{50}$ [57]	AP at IoU = 0.50	Basic localization capability
$AP_{75}$ [58]	AP at IoU = 0.75	Precise localization requirement
$AP_{S}$ [59]	AP for small targets (area < 1024 px^2^)	Small target performance (sub-32 × 32 px)
$AP_{M}$ [60]	AP for medium targets (1024 ≤ area ≤ 9216 px^2^)	Medium target detection (32 × 32 to 96 × 96px)
$AP_{L}$ [60]	AP for large targets (area > 9216 px^2^)	Oversized target capability

**Table 2:** Performance comparison of MCEM against state-of-the-art methods on SSDD dataset.

Methods	AP (%)	${\mathrm{AP}}_{50}$ (%)	${\mathrm{AP}}_{75}$ (%)	${\mathrm{AP}}_{S}$ (%)	${\mathrm{AP}}_{M}$ (%)	${\mathrm{AP}}_{L}$ (%)	P (%)	R (%)	F1 (%)	${\mathrm{mAP}}_{50}$ (%)
Faster-RCNN [42]	65.04	96.12	77.47	61.68	71.54	66.07	93.7	91.6	92.6	96.7
MobileNet [63]	41.88	79.89	38.97	35.65	56.90	29.09	74.2	71.5	72.8	80.4
SSD [62]	52.94	88.07	60.08	49.00	61.26	52.50	83.6	81.1	82.3	88.6
RetinaNet [61]	60.76	90.90	70.46	57.32	67.62	61.77	87.6	85.4	86.5	91.5
YOLOv7 [64]	56.80	89.90	64.30	55.00	63.30	32.20	86.7	84.3	85.5	90.5
YOLOv8 [65]	68.00	97.20	81.50	66.30	72.30	63.80	95.2	93.4	94.3	97.5
YOLOv10 [66]	67.00	96.20	80.30	65.20	71.60	51.20	93.5	91.4	92.4	96.8
YOLOv11 [67]	67.90	96.40	82.80	66.30	72.00	59.90	93.7	91.6	92.6	97.0
MCEM (Ours)	70.20	96.9	84.70	67.30	74.80	77.70	96.5	94.7	95.6	98.1

**Table 3:** Performance comparison of MCEM against state-of-the-art methods on HRSID dataset.

Methods	AP (%)	${\mathrm{AP}}_{50}$ (%)	${\mathrm{AP}}_{75}$ (%)	${\mathrm{AP}}_{S}$ (%)	${\mathrm{AP}}_{M}$ (%)	${\mathrm{AP}}_{L}$ (%)	P (%)	R (%)	F1 (%)	${\mathrm{mAP}}_{50}$ (%)
Faster-RCNN [42]	56.70	81.10	65.30	43.60	74.50	46.30	80.5	69.8	74.7	83.5
MobileNet [63]	35.40	59.10	39.00	16.10	62.00	34.60	57.6	51.4	54.3	61.2
SSD [62]	43.50	67.10	49.80	23.30	70.40	52.70	68.2	60.7	64.2	69.5
RetinaNet [61]	50.70	83.10	54.00	36.20	72.10	63.20	81.8	73.1	77.2	85.6
YOLOv7 [64]	54.00	85.40	58.60	40.50	73.20	70.00	84.0	75.5	79.5	87.8
YOLOv8 [65]	57.70	83.20	65.00	42.80	77.30	59.30	87.2	75.9	82.4	86.5
YOLOv10 [66]	57.30	83.80	65.10	43.80	75.80	41.70	82.5	73.9	77.9	86.2
YOLOv11 [67]	58.00	83.40	64.80	43.50	77.70	33.40	82.1	73.5	77.5	85.9
MCEM (Ours)	60.00	85.20	67.50	45.10	79.20	71.80	90.3	79.3	84.5	88.4

**Table 4:** Basic information of ablation experiment.

Case	FEM	F^2^M	DHM	P (%)	R (%)	${\mathrm{mAP}}_{50}$ (%)	${\mathrm{mAP}}_{50:95}$ (%)	F1 (%)	GFLOPs	Speed (ms)
Base	×	×	×	95.2	93.4	97.5	68.8	94.3	6.5	3.4
Case 1	✓	×	×	94.2	93.2	97.0	69.5	93.7	6.3	5.5
Case 2	×	✓	×	95.9	94.2	98.3	72.0	95.0	6.2	3.0
Case 3	×	×	✓	96.6	94.7	98.5	72.5	95.6	6.4	2.6
Case 4	✓	✓	×	97.0	94.7	98.8	72.1	95.8	6.0	3.0
Case 5	✓	✓	✓	96.5	94.7	98.1	73.5	95.6	6.1	2.9

**Table 5:** The situation under the COCO metrics of ablation experiment.

Case	AP (%)	${\mathrm{AP}}_{50}$ (%)	${\mathrm{AP}}_{75}$ (%)	${\mathrm{AP}}_{S}$ (%)	${\mathrm{AP}}_{M}$ (%)	${\mathrm{AP}}_{L}$ (%)
Base	68.3	96.8	80.3	65.4	73.8	66.6
Case 1	69.4	96.5	84.2	66.1	75.8	69.1
Case 2	69.7	97.2	83.4	66.5	74.6	73.8
Case 3	70.3	97.3	84.4	67.5	74.8	76.6
Case 4	69.6	97.9	82.9	66.5	75.1	72.3
Case 5	70.2	96.9	84.7	67.3	74.8	77.7

## Data Availability

The data presented in this study are available on request from the corresponding author.

## References

[1] Crisp D.J. (2004). The State-of-the-Art in Ship Detection in Synthetic Aperture RADAR Imagery.

[2] Zhang T., Ji J., Li X., Yu W., Xiong H. (2019). Ship detection from PolSAR imagery using the complete polarimetric covariance difference matrix. IEEE Trans. Geosci. Remote Sens..

[3] Xu W., Guo Z., Huang P., Tan W., Gao Z. (2025). Towards Efficient SAR Ship Detection: Multi-Level Feature Fusion and Lightweight Network Design. Remote Sens..

[4] Zhang T., Wang W., Yang Z., Yin J., Yang J. (2021). Ship detection from PolSAR imagery using the hybrid polarimetric covariance matrix. IEEE Geosci. Remote Sens. Lett..

[5] Zhang T., Yang Z., Gan H., Xiang D., Zhu S., Yang J. (2020). PolSAR ship detection using the joint polarimetric information. IEEE Trans. Geosci. Remote Sens..

[6] Tian Z., Wang W., Zhou K., Song X., Shen Y., Liu S. (2024). Weighted Pseudo-Labels and Bounding Boxes for Semisupervised SAR Target Detection. IEEE J. Sel. Top. Appl. Earth Obs. Remote Sens..

[7] Wang J., Quan S., Xing S., Li Y., Wu H., Meng W. (2025). PSO-based fine polarimetric decomposition for ship scattering characterization. ISPRS J. Photogramm. Remote Sens..

[8] Xie N., Zhang T., Zhang L., Chen J., Wei F., Yu W. (2025). VLF-SAR: A Novel Vision-Language Framework for Few-shot SAR Target Recognition. IEEE Trans. Circuits Syst. Video Technol..

[9] Robey F.C., Fuhrmann D.R., Kelly E.J., Nitzberg R. (1992). A CFAR adaptive matched filter detector. IEEE Trans. Aerosp. Electron. Syst..

[10] Wackerman C.C., Friedman K.S., Pichel W.G., Clemente-Colón P., Li X. (2001). Automatic detection of ships in RADARSAT-1 SAR imagery. Can. J. Remote Sens..

[11] Liu T., Yang Z., Yang J., Gao G. (2016). CFAR ship detection methods using compact polarimetric SAR in a K-Wishart distribution. IEEE J. Sel. Top. Appl. Earth Obs. Remote Sens..

[12] Chen J., Niu L., Zhang J., Si J., Qian C., Zhang L. Amodal instance segmentation via prior-guided expansion. Proceedings of the AAAI Conference on Artificial Intelligence.

[13] Huang B., Zhang T., Quan S., Wang W., Guo W., Zhang Z. (2025). Scattering Enhancement and Feature Fusion Network for Aircraft Detection in SAR Images. IEEE Trans. Circuits Syst. Video Technol..

[14] Girshick R. Fast R-CNN. Proceedings of the IEEE International Conference on Computer Vision (ICCV).

[15] Ren S., He K., Girshick R., Sun J. Faster R-CNN: Towards real-time object detection with region proposal networks. Proceedings of the International Conference on Neural Information Processing Systems.

[16] Chen S., Wang H., Xu F., Jin Y.-Q. (2016). Target classification using the deep convolutional networks for SAR images. IEEE Trans. Geosci. Remote Sens..

[17] Liu W., Anguelov D., Erhan D., Szegedy C., Reed S., Fu C.-Y., Berg A.C., Leibe B., Matas J., Sebe N., Welling M. (2016). SSD: Single shot multibox detector. Computer Vision–ECCV 2016.

[18] Redmon J., Divvala S., Girshick R., Farhadi A. You only look once: Unified, real-time object detection. Proceedings of the IEEE Conference on Computer Vision and Pattern Recognition (CVPR).

[19] Chen J., Yan J., Fang Y., Niu L. (2025). Webly supervised fine-grained classification by integrally tackling noises and subtle differences. IEEE Trans. Image Process..

[20] He K., Zhang X., Ren S., Sun J. Deep Residual Learning for Image Recognition. Proceedings of the IEEE Conference on Computer Vision and Pattern Recognition (CVPR).

[21] Cui Z., Wang X., Liu N., Cao Z., Yang J. (2021). Ship Detection in Large-Scale SAR Images Via Spatial Shuffle-Group Enhance Attention. IEEE Trans. Geosci. Remote Sens..

[22] Zhu M., Hu G., Li S., Zhou H., Wang S., Feng Z. (2022). A Novel Anchor-Free Method Based on FCOS + ATSS for Ship Detection in SAR Images. Remote Sens..

[23] Chen Y., Zhu X., Li Y., Wei Y., Ye L. (2023). Enhanced Semantic Feature Pyramid Network for Small Object Detection. Signal Process. Image Commun..

[24] Wang Z., Wang C., Pei J., Huang Y., Zhang Y., Yang H. A Deformable Convolution Neural Network for SAR ATR. Proceedings of the IGARSS 2020—IEEE International Geoscience and Remote Sensing Symposium.

[25] Chen P., Zhou H., Li Y., Liu P., Liu B. (2023). A Novel Deep Learning Network with Deformable Convolution and Attention Mechanisms for Complex Scenes Ship Detection in SAR Images. Remote Sens..

[26] Panda S.L., Sahoo U.K., Maiti S., Sasmal P. (2024). An Attention U-Net-Based Improved Clutter Suppression in GPR Images. IEEE Trans. Instrum. Meas..

[27] Li Z., He H., Zhou T., Zhang Q., Han X., You Y. (2025). Dual CG-IG Distribution Model for Sea Clutter and Its Parameter Correction Method. J. Syst. Eng. Electron..

[28] Li M., Lin S., Huang X. SAR Ship Detection Based on Enhanced Attention Mechanism. Proceedings of the 2021 2nd International Conference on Artificial Intelligence and Computer Engineering (ICAICE).

[29] Li Y., Liu J., Li X., Zhang X., Wu Z., Han B. (2025). A Lightweight Network for Ship Detection in SAR Images Based on Edge Feature Aware and Fusion. IEEE J. Sel. Top. Appl. Earth Obs. Remote Sens..

[30] Zhang Y., Cai W., Guo J., Kong H., Huang Y., Ding X. (2025). Lightweight SAR Ship Detection via Pearson Correlation and Nonlocal Distillation. IEEE Geosci. Remote Sens. Lett..

[31] Liu M., Xu J., Zhou Y. Real-time processing on airborne platforms. Proceedings of the IEEE/CVF Conference on Computer Vision and Pattern Recognition Workshops (CVPRW).

[32] Zhang Y., Ye M., Zhu G., Liu Y., Guo P., Yan J. (2024). FFCA-YOLO for small object detection in remote sensing images. IEEE Trans. Geosci. Remote Sens..

[33] Kim K.-H., Hong S., Roh B., Cheon Y., Park M. (2016). PVANET: Deep but lightweight neural networks for real-time object detection. arXiv.

[34] He F., Wang C., Guo B. (2025). SSGY: A Lightweight Neural Network Method for SAR Ship Detection. Remote Sens..

[35] Sunkara R., Luo T., Amini M.-R., Canu S., Fischer A., Guns T., Kralj Novak P., Tsoumakas G. (2023). No More Strided Convolutions or Pooling: A New CNN Building Block for Low-Resolution Images and Small Objects. Machine Learning and Knowledge Discovery in Databases.

[36] Kang M., Ting C.-M., Ting F.F., Phan R.C.-W., Greenspan H., Madabhushi A., Mousavi P., Salcudean S., Duncan J., Syeda-Mahmood T., Taylor R. (2023). RCS-YOLO: A Fast and High-Accuracy Object Detector for Brain Tumor Detection. Medical Image Computing and Computer Assisted Intervention—MICCAI 2023.

[37] Zhao Y., Lv W., Xu S., Wei J., Wang G., Dang Q., Liu Y., Chen J. (2024). DETRs beat YOLOs on real-time object detection. arXiv.

[38] Wei S., Zeng X., Qu Q., Wang M., Su H., Shi J. (2020). HRSID: A high-resolution SAR images dataset for ship detection and instance segmentation. IEEE Access.

[39] Zhang T., Zhang X., Li J., Xu X., Wang B., Zhan X., Xu Y., Ke X., Zeng T., Su H. (2021). SAR Ship Detection Dataset (SSDD): Official Release and Comprehensive Data Analysis. Remote Sens..

[40] Tian Z., Shen C., Chen H., He T. FCOS: Fully convolutional one-stage object detection. Proceedings of the IEEE International Conference on Computer Vision (ICCV).

[41] Meng S., Ren K., Lu D., Gu G., Chen Q., Lu G. (2018). A Novel Ship CFAR Detection Algorithm Based on Adaptive Parameter Enhancement and Wake-Aided Detection in SAR Images. Infrared Phys. Technol..

[42] Li Y., Zhang S., Wang W.-Q. (2019). A lightweight faster R-CNN for ship detection in SAR images. IEEE Geosci. Remote Sens. Lett..

[43] Xu P., Li Q., Zhang B., Wu F., Zhao K., Du X., Yang C., Zhong R. (2021). On-Board Real-Time Ship Detection in HISEA-1 SAR Images Based on CFAR and Lightweight Deep Learning. Remote Sens..

[44] Lin T.-Y., Dollár P., Girshick R., He K., Hariharan B., Belongie S. Feature Pyramid Networks for Object Detection. Proceedings of the 2017 IEEE Conference on Computer Vision and Pattern Recognition (CVPR).

[45] Liu S., Qi L., Qin H., Shi J., Jia J. Path Aggregation Network for Instance Segmentation. Proceedings of the 2018 IEEE/CVF Conference on Computer Vision and Pattern Recognition (CVPR).

[46] Tan M., Pang R., Le Q.V. EfficientDet: Scalable and Efficient Object Detection. Proceedings of the 2020 IEEE/CVF Conference on Computer Vision and Pattern Recognition (CVPR).

[47] Gao J., Geng X., Zhang Y., Wang R., Shao K. (2024). Augmented Weighted Bidirectional Feature Pyramid Network for Marine Object Detection. Expert Syst. Appl..

[48] Woo S., Park J., Lee J.-Y., Kweon I.S. (2018). CBAM: Convolutional Block Attention Module. arXiv.

[49] Hu J., Shen L., Sun G. Squeeze-and-Excitation Networks. Proceedings of the 2018 IEEE/CVF Conference on Computer Vision and Pattern Recognition (CVPR).

[50] Chen L.-C., Zhu Y., Papandreou G., Schroff F., Adam H., Ferrari V., Hebert M., Sminchisescu C., Weiss Y. (2018). Encoder-Decoder with Atrous Separable Convolution for Semantic Image Segmentation. Computer Vision—ECCV 2018.

[51] Dosovitskiy A., Beyer L., Kolesnikov A., Weissenborn D., Zhai X., Unterthiner T., Dehghani M., Minderer M., Heigold G., Gelly S. (2020). An Image is Worth 16 × 16 Words: Transformers for Image Recognition at Scale. arXiv.

[52] Feng C., Zhong Y., Gao Y., Scott M.R., Huang W. TOOD: Task-aligned One-stage Object Detection. Proceedings of the 2021 IEEE/CVF International Conference on Computer Vision (ICCV).

[53] Wu Y., He K. Group normalization. Proceedings of the European Conference on Computer Vision (ECCV).

[54] Manning C.D., Raghavan P., Schütze H. (2008). Introduction to Information Retrieval.

[55] Li L., Ma H., Zhang X., Zhao X., Lv M., Jia Z. (2024). Synthetic Aperture Radar Image Change Detection Based on Principal Component Analysis and Two-Level Clustering. Remote Sens..

[56] Qian Y., Yan S., Lukežič A., Kristan M., Kämäräinen J.-K., Matas J. DAL: A Deep Depth-Aware Long-term Tracker. Proceedings of the 2020 25th International Conference on Pattern Recognition (ICPR).

[57] Everingham M., Van Gool L., Williams C.K.I., Winn J., Zisserman A. (2010). The Pascal Visual Object Classes (VOC) Challenge. Int. J. Comput. Vis..

[58] Lin T.-Y., Maire M., Belongie S., Hays J., Perona P., Ramanan D., Dollár P., Zitnick C.L. (2014). Microsoft COCO: Common Objects in Context. Proceedings of the European Conference on Computer Vision–ECCV 2014.

[59] Zhang W., Wang S., Thachan S., Chen J., Qian Y. Deconv R-CNN for Small Object Detection on Remote Sensing Images. Proceedings of the 2018 IEEE International Geoscience and Remote Sensing Symposium (IGARSS).

[60] Liu L., Pan Z., Lei B. (2017). Learning a Rotation Invariant Detector with Rotatable Bounding Box. arXiv.

[61] Li Q., Xiao D., Shi F. (2022). A decoupled head and coordinate attention detection method for ship targets in SAR images. IEEE Access.

[62] Yue T., Zhang Y., Liu P., Xu Y., Yu C. (2022). A generating-anchor network for small ship detection in SAR images. IEEE J. Sel. Top. Appl. Earth Obs. Remote Sens..

[63] Hao Y., Zhang Y. (2024). A lightweight convolutional neural network for ship target detection in SAR images. IEEE Trans. Aerosp. Electron. Syst..

[64] Zhou L., Wan Z., Zhao S., Han H., Liu Y. (2024). BFEA: A SAR ship detection model based on attention mechanism and multiscale feature fusion. IEEE J. Sel. Top. Appl. Earth Obs. Remote Sens..

[65] Zhao L., Ning F., Xi Y., Liang G., He Z., Zhang Y. (2024). MSFA-YOLO: A multi-scale SAR ship detection algorithm based on fused attention. IEEE Access.

[66] Wang Z., Miao F., Huang Y., Lu Z., Ohtsuki T., Gui G. Object Detection on SAR Images Via YOLOv10 and Integrated ACmix Attention Mechanism. Proceedings of the 2024 6th International Conference on Robotics, Intelligent Control and Artificial Intelligence (RICAI).

[67] Bakirci M., Bayraktar I. Assessment of YOLO11 for Ship Detection in SAR Imagery Under Open Ocean and Coastal Challenges. Proceedings of the 2024 21st International Conference on Electrical Engineering, Computing Science and Automatic Control (CCE).

